# Supplementary material for: Evidence of non-tandemly repeated rDNAs and their intragenomic heterogeneity in Rhizophagus irregularis
Source: Commun Biol. 2018 Jul 10;1:87. doi: 10.1038/s42003-018-0094-7 (PMC6123716; doi:10.1038/s42003-018-0094-7)
Supplement: Supplementary file 1 — Supplementary Information [file 42003_2018_94_MOESM1_ESM.pdf]

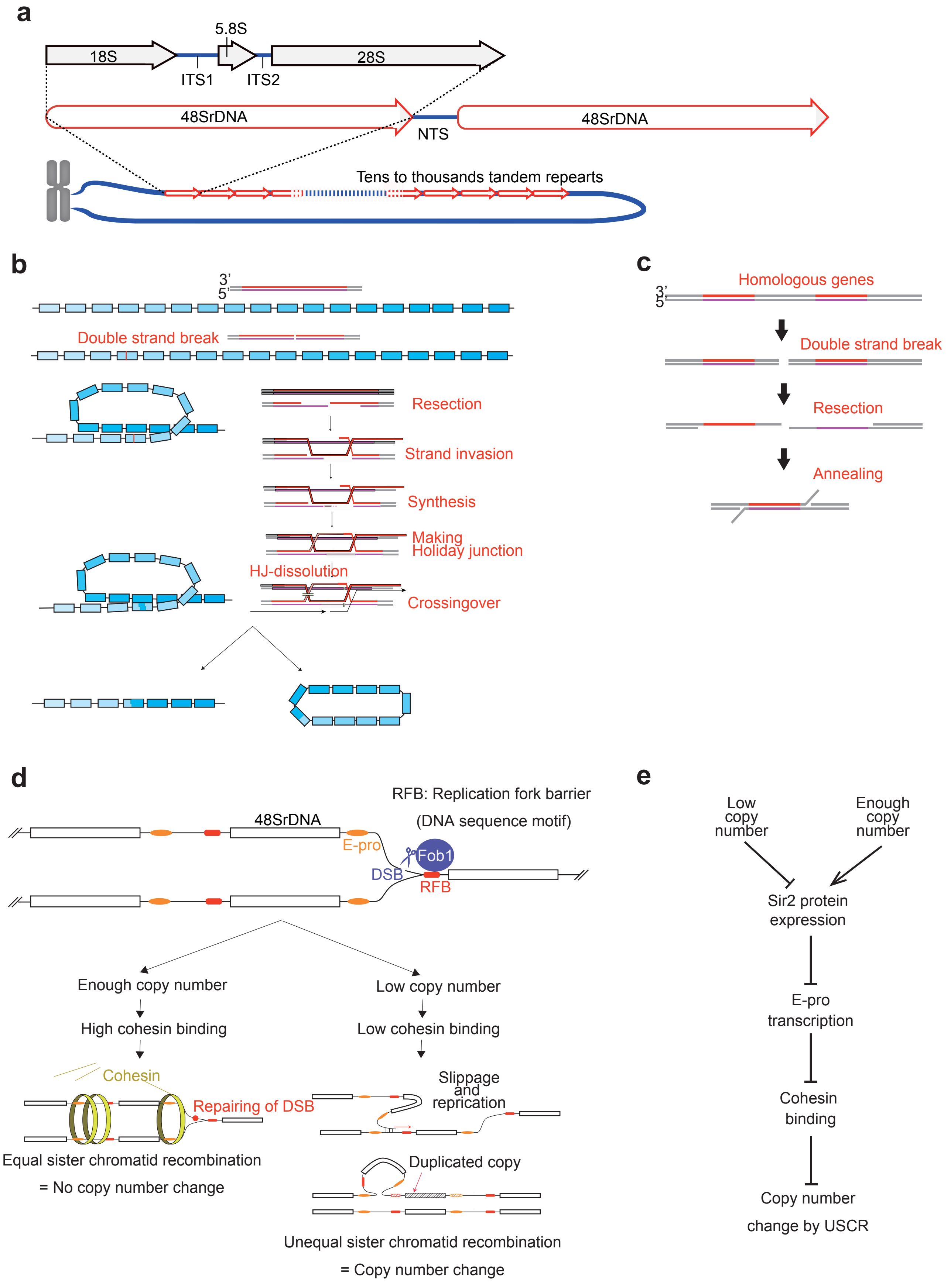

Supplementary Figure 1. General concerted evolution of eukaryotic rDNA

a. A general structure of eukaryotic rDNA clusters[20]. **b**. Deletion of homologous genes by crossover recombination[33,58]. **c**. Deletion of homologous genes by single-strand annealing[34]. **d**. A model of the rDNA number maintenance system[32]. **e**. Copy number-controlling pathway in yeast[32].

Gene models excluede “Provisional” models

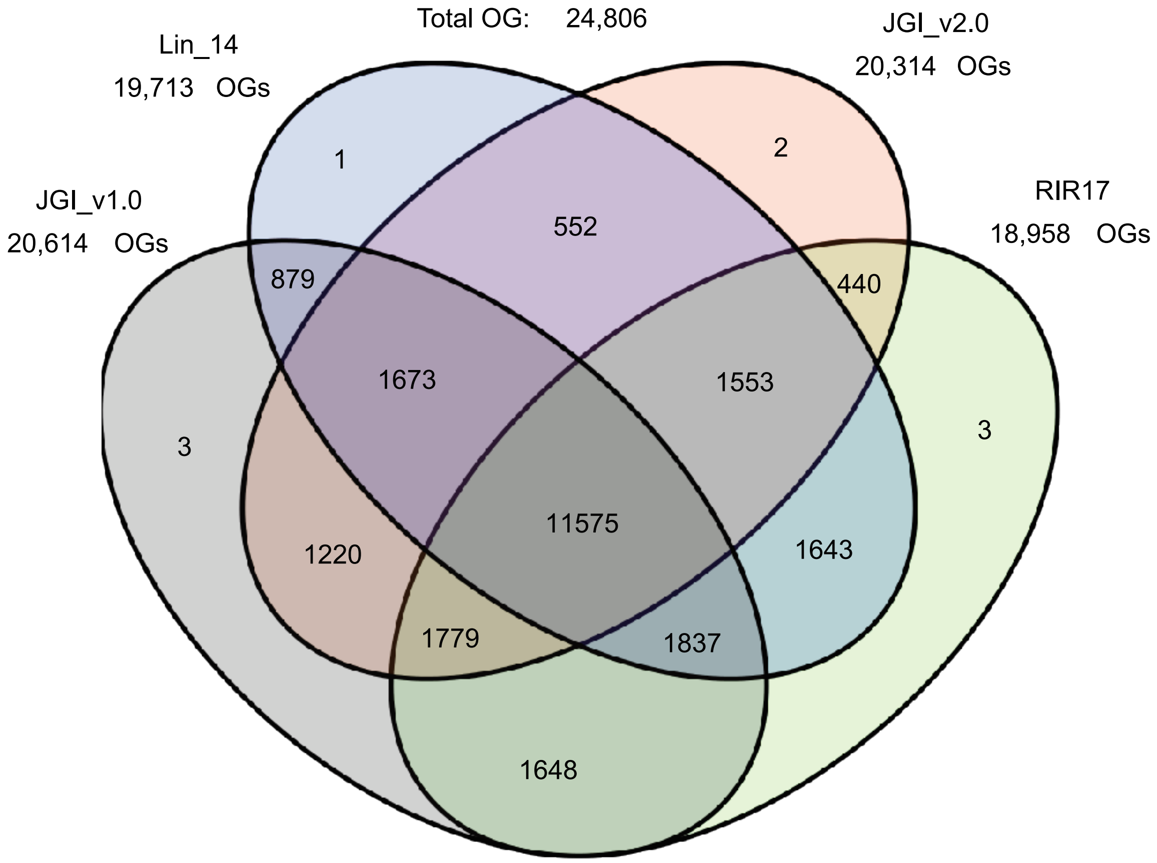

All gene medels including “Provisional” models

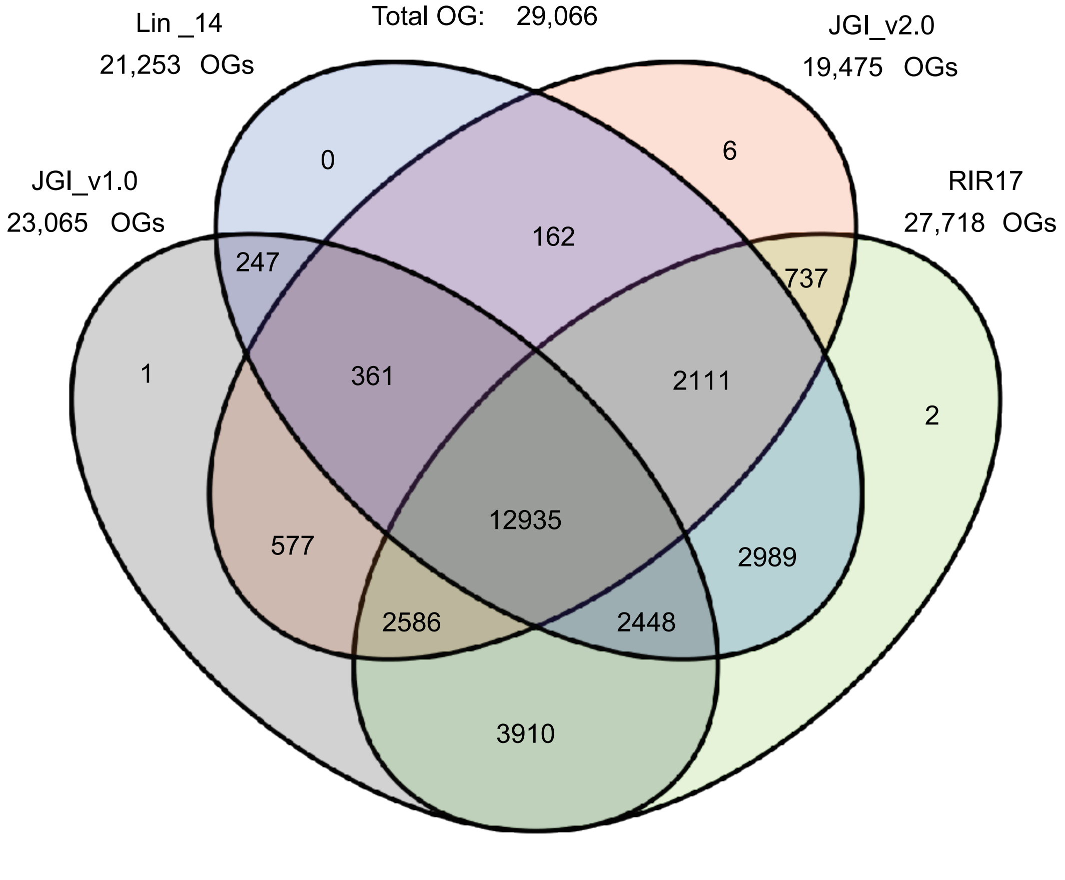

Supplementary Figure 2. Cross comparison of *R. irregularis* DAOM-18160219 orthologous genes from three genomic studies and our RIR17 assemblies.

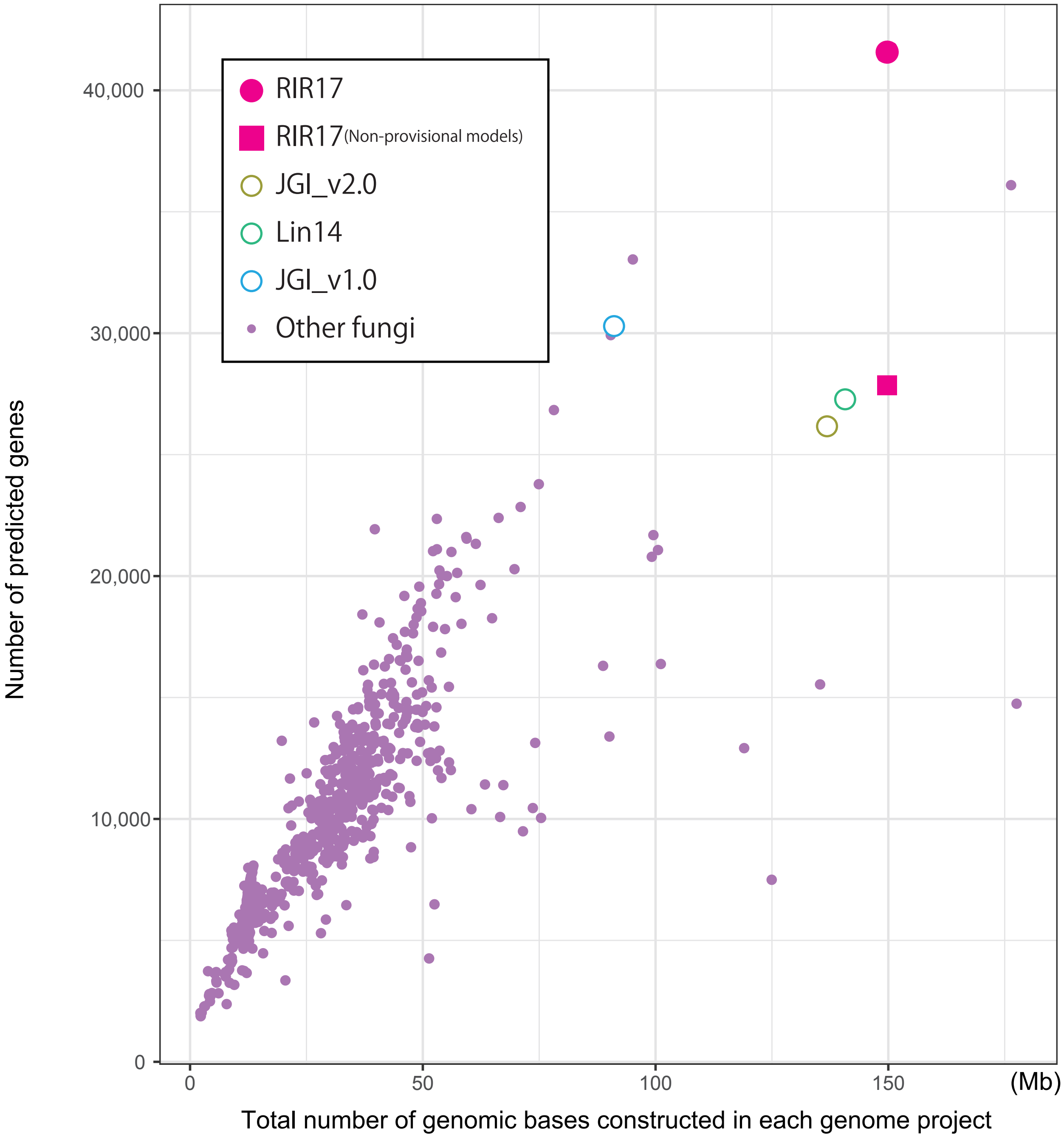

Supplementary Figure 3. Total assembly size and predicted gene number in fungi. *Rhizophagus irregularis* genomes (RIR17 and three previously assembled genomes, JGI_v1.0, JGI_v2.0 and Lin14) and 768 genomes registered in GenBank. The fungal assembly statistics were obtained from the registered information in GenBank (ftp://ftp.ncbi.nlm.nih.gov/genomes/ASSEMBLY_REPORTS/All/).

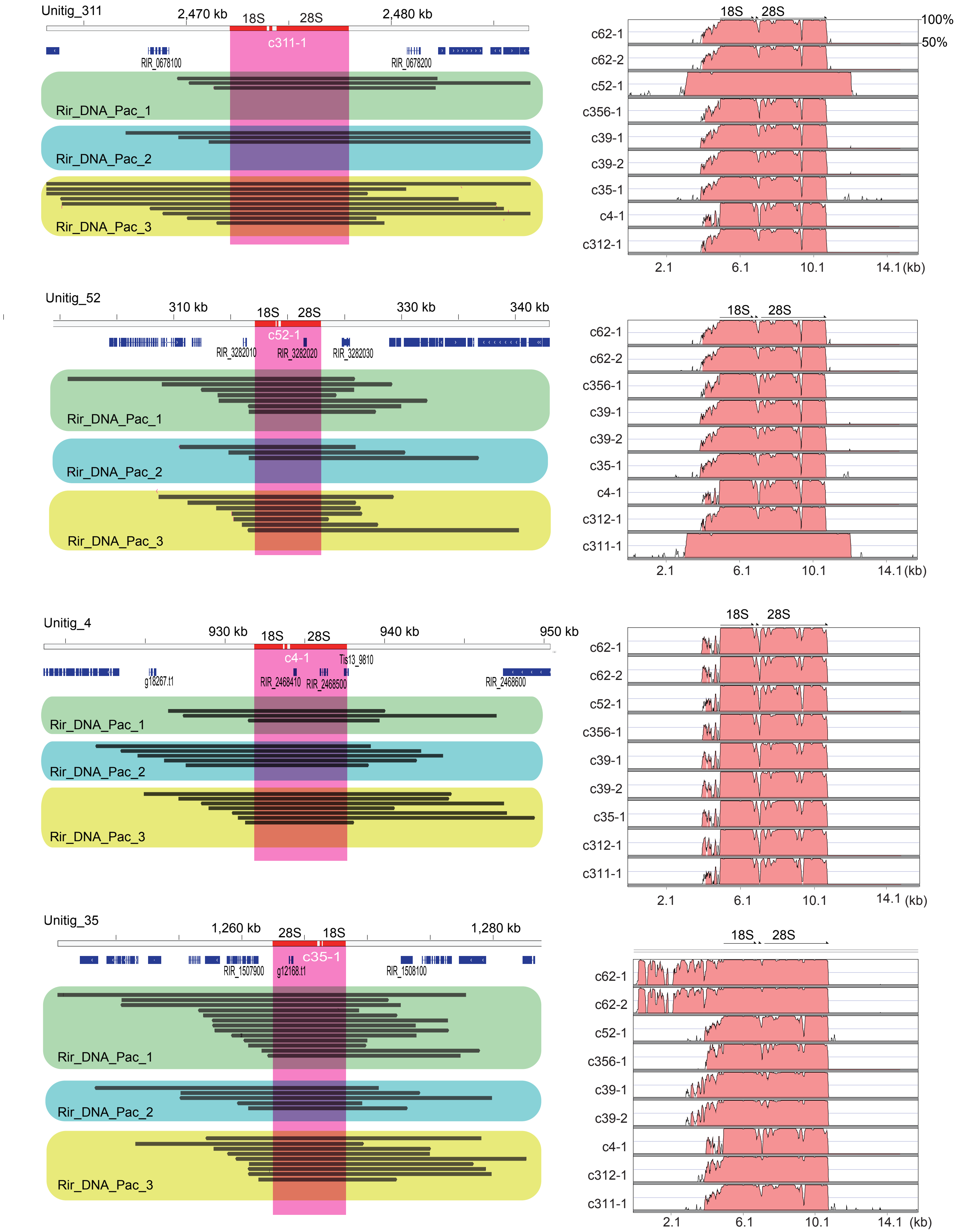

Unitig_311
2,470 kb
18S
28S
2,480 kb
c311-1
RIR_0678100
RIR_0678200
Rir_DNA_Pac_1
Rir_DNA_Pac_2
Rir_DNA_Pac_3
18S
28S
100%
50%
c62-1
c62-2
c52-1
c356-1
c39-1
c39-2
c35-1
c4-1
c312-1
2.1
6.1
10.1
14.1 (kb)
Unitig_52
310 kb
18S
28S
330 kb
340 kb
c52-1
RIR_3282010
RIR_3282020
RIR_3282030
Rir_DNA_Pac_1
Rir_DNA_Pac_2
Rir_DNA_Pac_3
18S
28S
c62-1
c62-2
c356-1
c39-1
c39-2
c35-1
c4-1
c312-1
c311-1
2.1
6.1
10.1
14.1 (kb)
Unitig_4
930 kb
18S
28S
940 kb
950 kb
c4-1
Tis13_9810
g18267.t1
RIR_2468410
RIR_2468500
RIR_2468600
Rir_DNA_Pac_1
Rir_DNA_Pac_2
Rir_DNA_Pac_3
18S
28S
c62-1
c62-2
c52-1
c356-1
c39-1
c39-2
c35-1
c312-1
c311-1
2.1
6.1
10.1
14.1 (kb)
Unitig_35
1,260 kb
28S
18S
1,280 kb
c35-1
RIR_1507900
g12168.t1
RIR_1508100
Rir_DNA_Pac_1
Rir_DNA_Pac_2
Rir_DNA_Pac_3
18S
28S
c62-1
c62-2
c52-1
c356-1
c39-1
c39-2
c4-1
c312-1
c311-1
2.1
6.1
10.1
14.1 (kb)

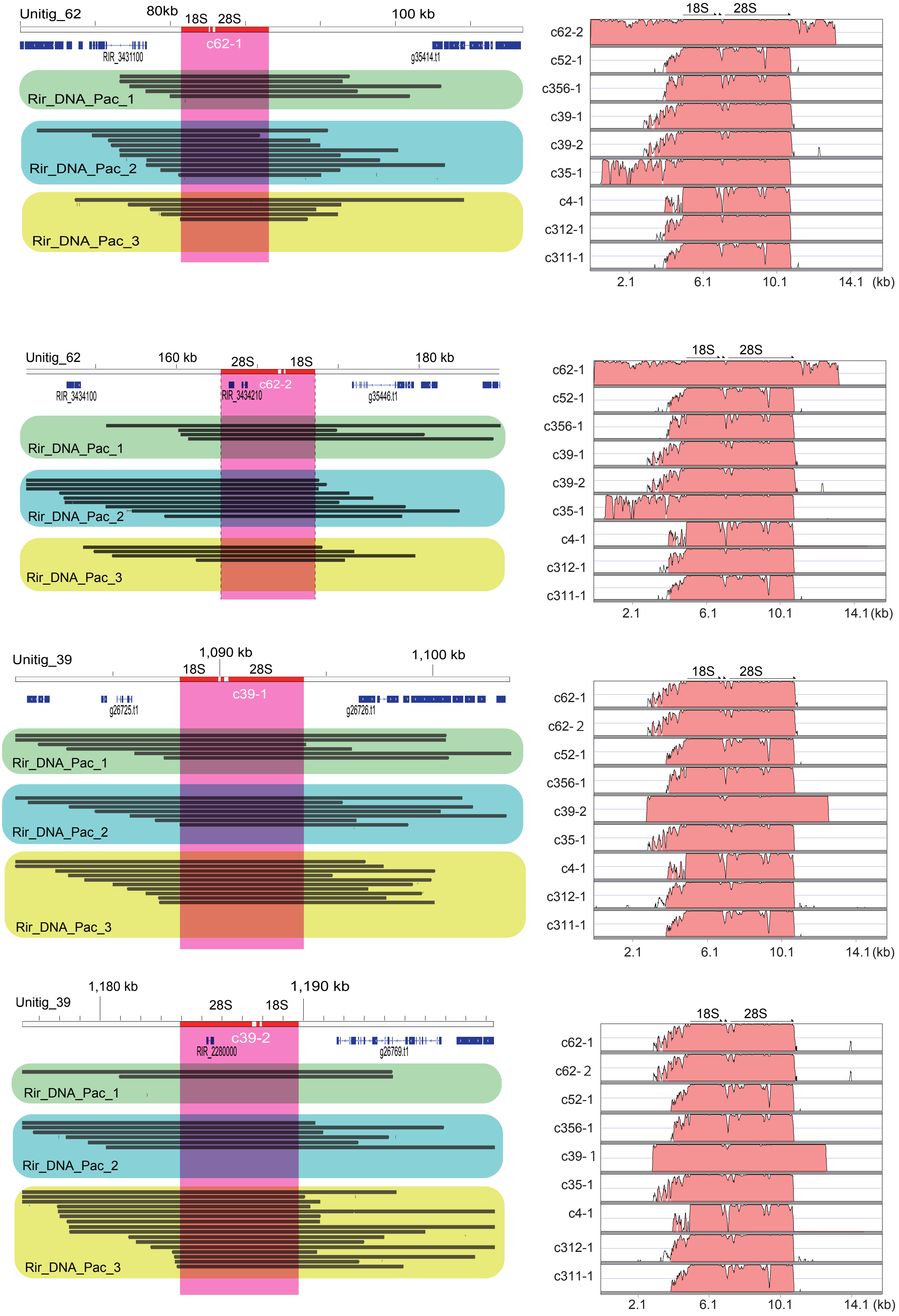

Unitig_62
80kb
100 kb
18S
28S
c62-1
RIR_3431100
g35414.t1
Rir_DNA_Pac_1
Rir_DNA_Pac_2
Rir_DNA_Pac_3
c62-2
c52-1
c356-1
c39-1
c39-2
c35-1
c4-1
c312-1
c311-1
2.1
6.1
10.1
14.1
(kb)
Unitig_62
160 kb
180 kb
28S
18S
c62-2
RIR_3434100
RIR_3434210
g35446.t1
Rir_DNA_Pac_1
Rir_DNA_Pac_2
Rir_DNA_Pac_3
c62-1
c52-1
c356-1
c39-1
c39-2
c35-1
c4-1
c312-1
c311-1
2.1
6.1
10.1
14.1 (kb)
Unitig_39
1,090 kb
1,100 kb
18S
28S
c39-1
g26725.t1
g26726.t1
Rir_DNA_Pac_1
Rir_DNA_Pac_2
Rir_DNA_Pac_3
c62-1
c62- 2
c52-1
c356-1
c39-2
c35-1
c4-1
c312-1
c311-1
2.1
6.1
10.1
14.1 (kb)
Unitig_39
1,180 kb
1,190 kb
28S
18S
c39-2
RIR_2280000
g26769.t1
Rir_DNA_Pac_1
Rir_DNA_Pac_2
Rir_DNA_Pac_3
c62-1
c62- 2
c52-1
c356-1
c39- 1
c35-1
c4-1
c312-1
c311-1
2.1
6.1
10.1
14.1 (kb)

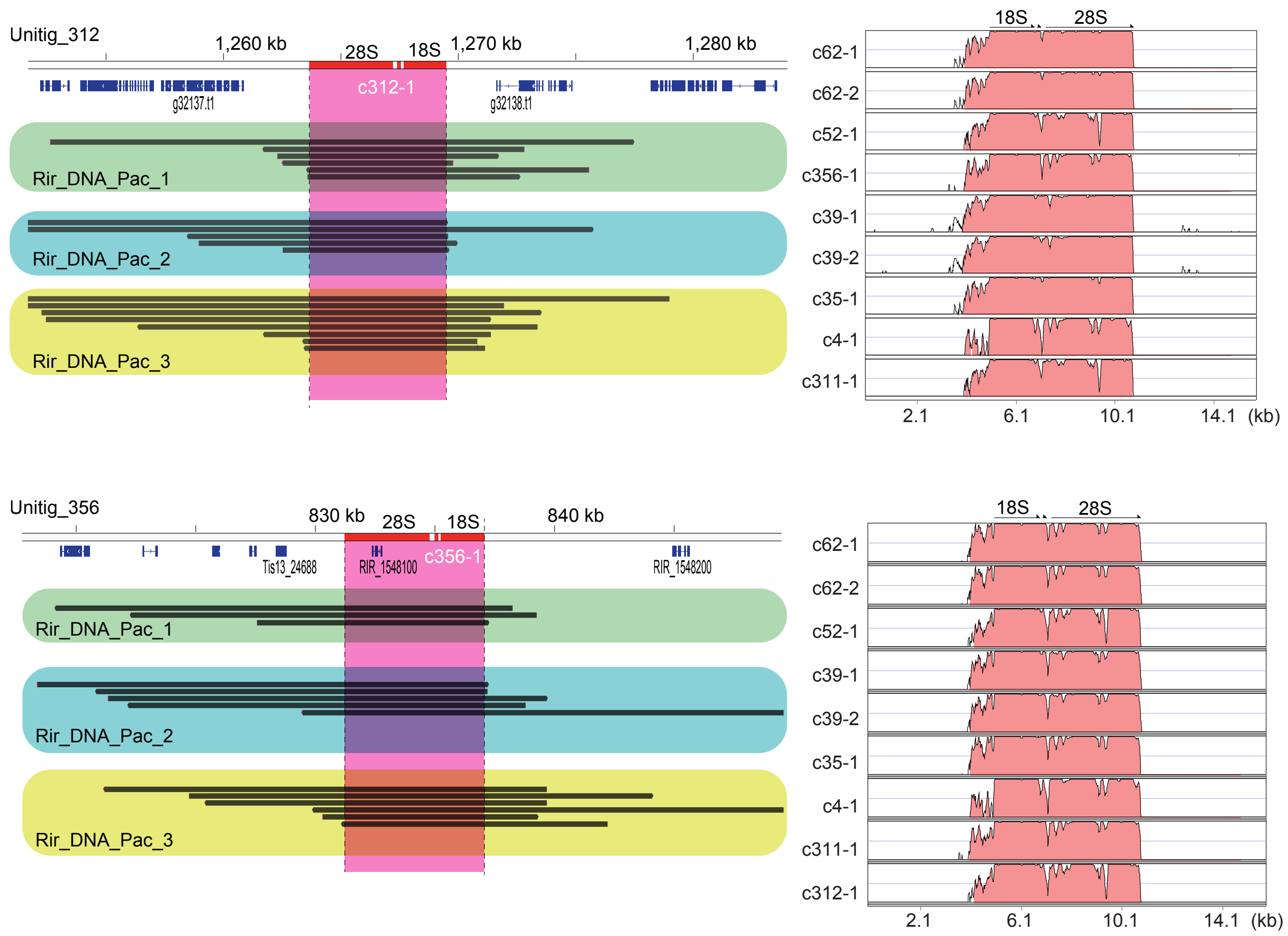

Supplementary Figure 4. Mapped PacBio reads of rDNA regions of RIR17 contigs and the sequence similarity of rDNAs with other rDNA regions in RIR17

The colors have the same meanings as in Fig. 3a-b.

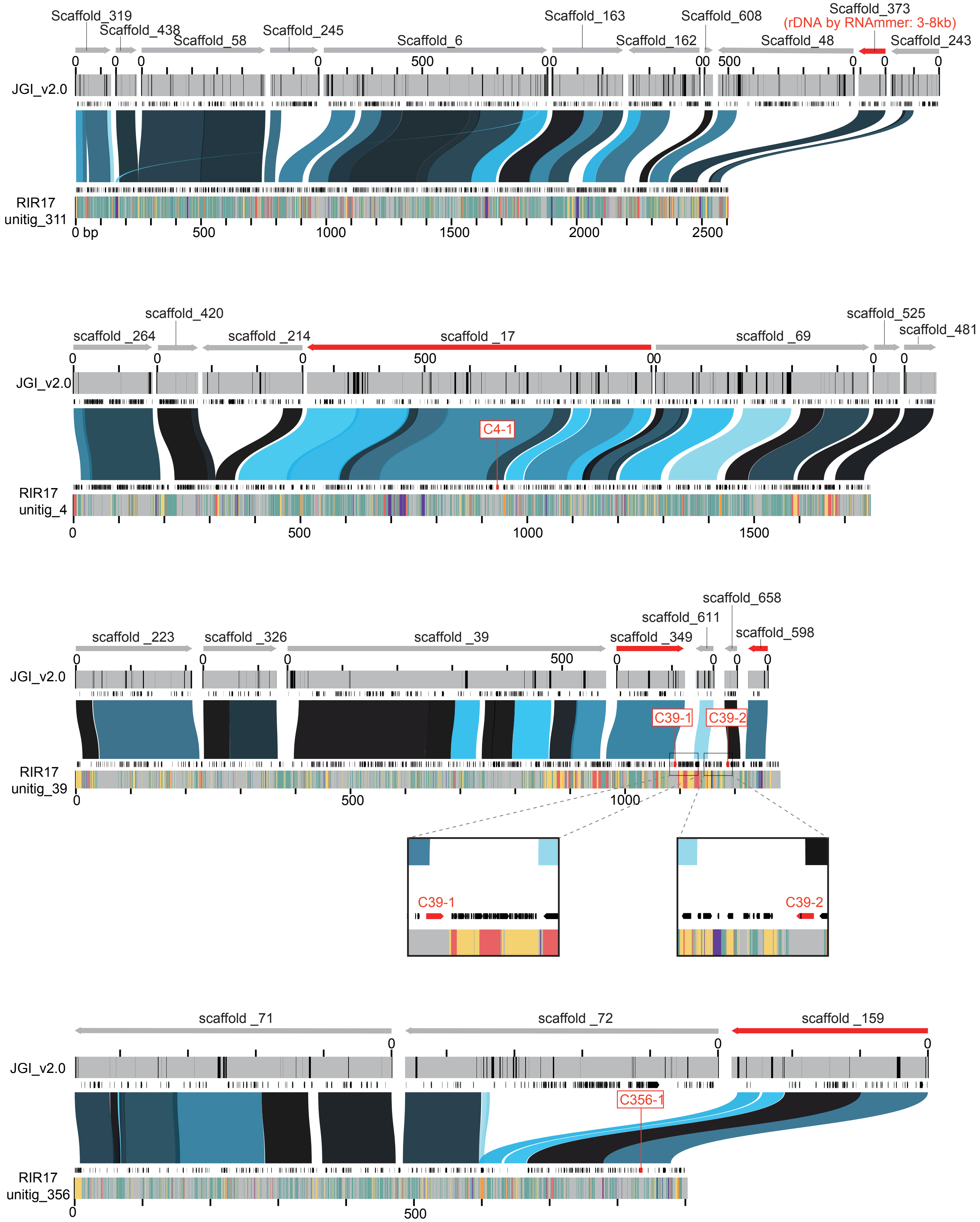

Scaffold_319
Scaffold_438
Scaffold_58
Scaffold_245
Scaffold_6
Scaffold_163
Scaffold_162
Scaffold_608
Scaffold_48
Scaffold_373
(rDNA by RNAmmer: 3-8kb)
Scaffold_243
JGI_v2.0
RIR17
unitig_311
0 bp
500
1000
1500
2000
2500
scaffold_264
scaffold_420
scaffold_214
scaffold_17
scaffold_69
scaffold_525
scaffold_481
C4-1
RIR17
unitig_4
scaffold_223
scaffold_326
scaffold_39
scaffold_349
scaffold_611
scaffold_658
scaffold_598
C39-1
C39-2
RIR17
unitig_39
scaffold_71
scaffold_72
scaffold_159
C356-1
RIR17
unitig_356

(Continued)

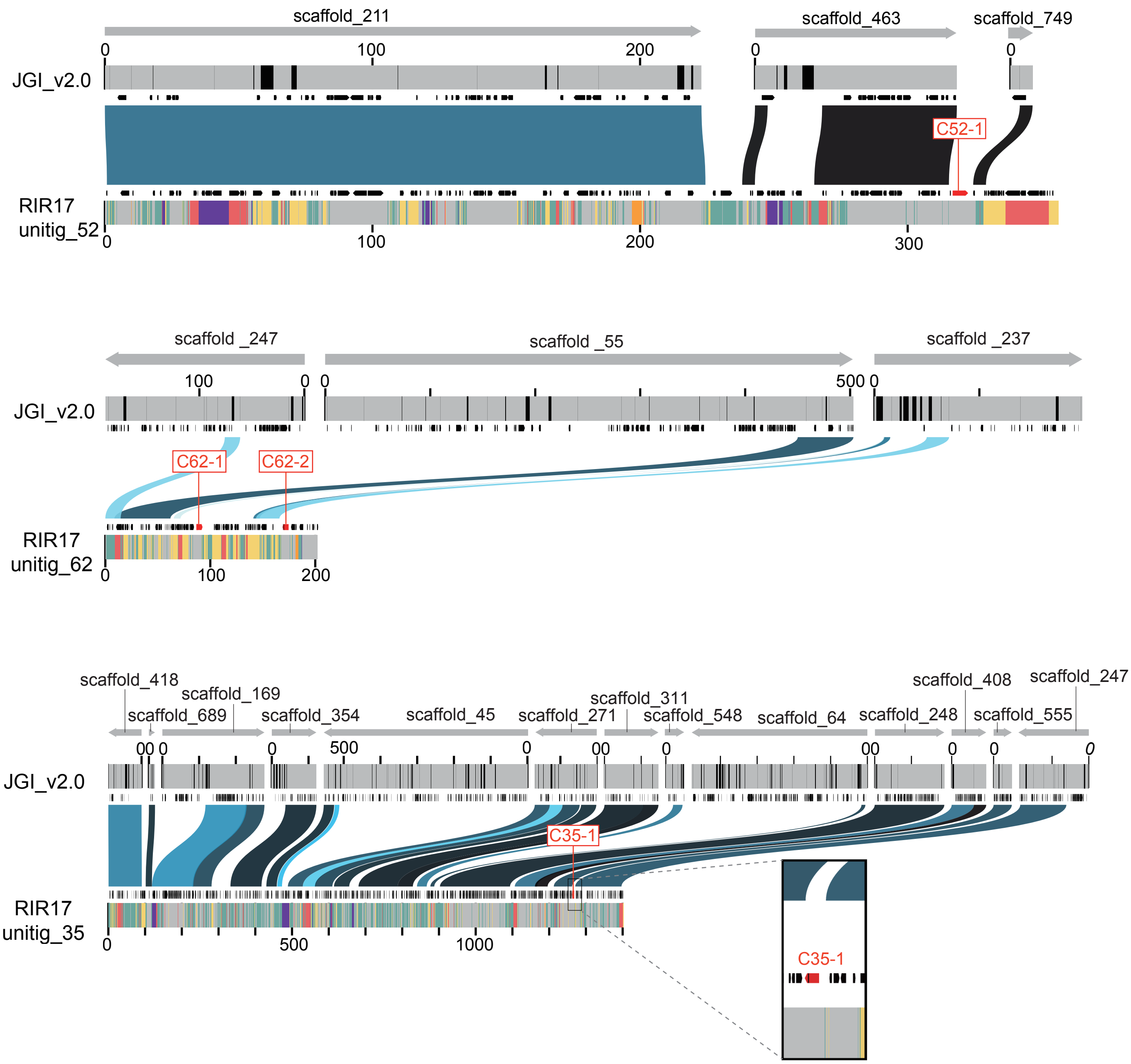

Supplementary Figure 5. Positions and identities of JGI_v2.0 scaffold aligned against RIR17 contigs with rDNAs.

The colors have the same meanings as in Figure 3c.

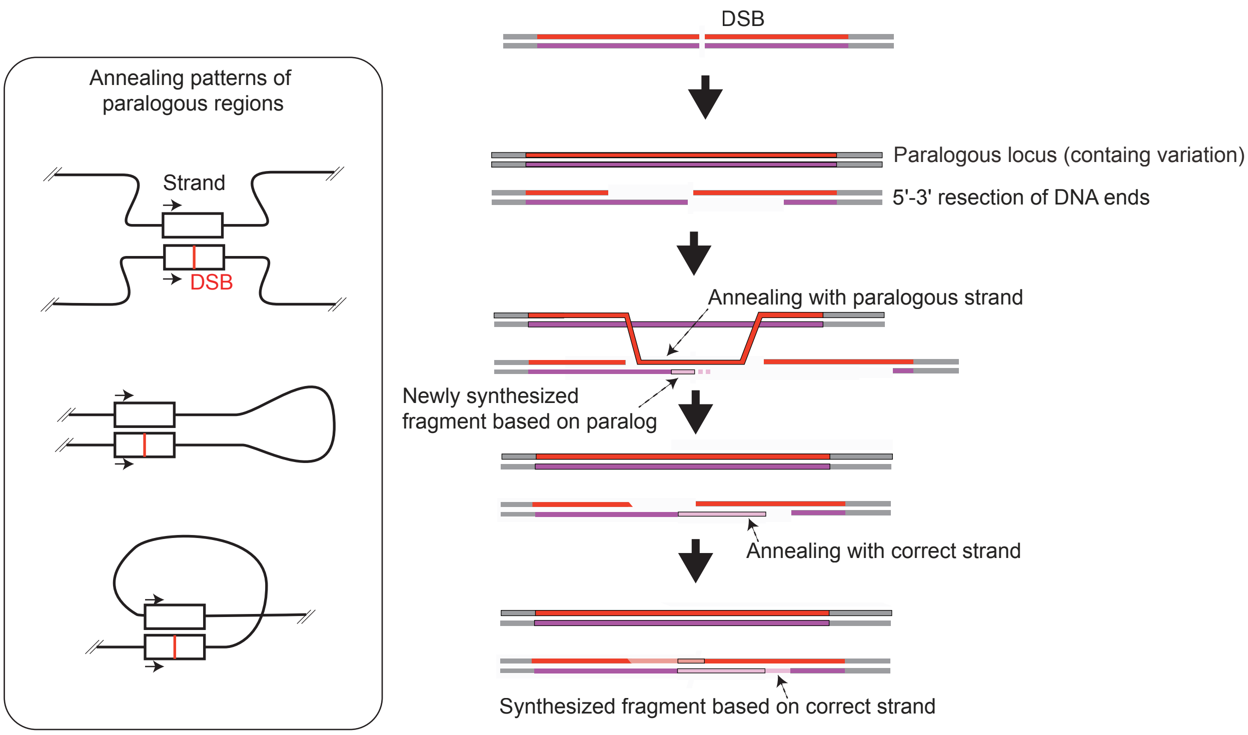

Supplementary Figure 6. Synthesis-dependent strand annealing

Supplementary Table 1. Assmbly statistics of *R. irregularis* genomes

Assmbly ID	Reference	IDs in public database (database)	# assembly	Total length	% total length	# N's base	N50 (bp)	Longest assembly	CEGMA completenss* Detected gene #	%
RIR17	-	BDIQ01000000 (INSDC)	210	149,750,837	97.24	0	2,308,146	5,727,599	244	98.4
JGI_v2.0	Chen et a. 2018	Rhizophagus irregulare v2.0 (MycoCosm)	1,123	136,807,476	88.84	6,925,426	336,373	1,375,864	245	98.8
Lin_14	Lin et al. 2014	JEMT00000000 (INSDC)	30,233	140,695,967	91.36	30,115	16,434	198,953	229	92.3
JGI_v1.0	Tisserant et al. 2013	Rhizophagus irregulare v1.0 (MycoCosm)	28,371	91,083,792	59.15	270,422	4,186	57,883	241	97.2

*All CEGMA restuls was obtained by our reanalysis on CEGMA

Supplementary Table 2. Summary of the sequencing data

Sequence ID	DRA ID	Libraly construction and sequencing method	DNA/RNA	Read #	Read length	Total basepair (Mbp)
Rir_DNA_Pac_1	DRA004849	PacBio RSII, P6-C4 sequence chemistry	DNA	348,539	*7559	2,635
Rir_DNA_Pac_2	DRA004878	PacBio RSII, P6-C4 sequence chemistry	DNA	394,261	*8562	3,376
Rir_DNA_Pac_3	DRA004889	PacBio RSII, P6-C4 sequence chemistry	DNA	761,112	*7500	5,708
Rir_DNA_PE180	DRA004835	Hiseq PE126,TruSeq DNA prep kit	DNA	423,041,682	126	53,303
Rir_RNA_SS	DRA005204	Hiseq PE101,TruSeq RNA prep kit	RNA	32,245,928	101	3,257
Rir_RNA_rRNA	DRA006039	Miseq PE301,TruSeq RNA prep kit	RNA	20,904,594	301	6,292

*Average read length

Supplementary Table 3. Blast hit data of eliminated contamination sequences

Query; Illumina-polished assmbly, Database; Refseq_genome, blast; default blastn search

Assmbly ID	Hit ID	Assmbly length	Identity %	Query cover %	Max bit score	Total score	E-value	Subject title
unitig_103	NW_016089427	4223	99	100	7548	25444	0	*Daucus carota* subsp. *sativus* cultivar DH1 unplaced genomic scaffold, ASM162521v1 DCARv2_B11, whole genome shotgun sequence
unitig_228	NC_030383	2192	93	100	3208	87603	0	*Daucus carota* subsp. *sativus* cultivar DH1 chromosome 3, ASM162521v1, whole genome shotgun sequence
unitig_85	NW_016092113	14490	99	99	10975	106600	0	*Daucus carota* subsp. *sativus* cultivar DH1 unplaced genomic scaffold, ASM162521v1 DCARv2_S316, whole genome shotgun sequence
unitig_109	NW_016089417	1602	99	100	2826	52864	0	*Daucus carota* subsp. *sativus* cultivar DH1 chromosome 2 genomic scaffold, ASM162521v1 2, whole genome shotgun sequence
unitig_88	NW_016092113	14867	98	100	11607	90386	0	*Daucus carota* subsp. *sativus* cultivar DH1 unplaced genomic scaffold, ASM162521v1 DCARv2_S316, whole genome shotgun sequence
unitig_100	NW_016089417	8259	94	100	12676	539700	0	*Daucus carota* subsp. *sativus* cultivar DH1 chromosome 2 genomic scaffold, ASM162521v1 2, whole genome shotgun sequence
unitig_83	NW_016092113	46670	98	100	11686	328700	0	*Daucus carota* subsp. *sativus* cultivar DH1 unplaced genomic scaffold, ASM162521v1 DCARv2_S316, whole genome shotgun sequence
unitig_84	NW_016092113	14454	98	100	11673	103600	0	*Daucus carota* subsp. *sativus* cultivar DH1 unplaced genomic scaffold, ASM162521v1 DCARv2_S316, whole genome shotgun sequence
unitig_101	NC_030388	3879	95	100	6058	146300	0	*Daucus carota* subsp. *sativus* cultivar DH1 chromosome 8, ASM162521v1, whole genome shotgun sequence

Supplementary Table 4. Statisticsof Mummer alignment

			RIR17 vs JGI2		RIR17 vs Lin14		RIR17 vs JGI_v1.0		JGI_v2.0 vs Lin14		JGI_v2.0 vs JGI_v1.0	
			RIR17	JGI_v2.0	RIR17	Lin14	RIR17	JGI_v1.0	JGI_v2.0	Lin14	JGI_v2.0	JGI_v1.0
Assmbly #	Total query		210	1,123	210	30233	210	28,371	1,123	30,233	1,123	28,371
	Aligned		181	1,118	184	29,346	171	27,941	1,097	29,222	1,039	27,911
	Unaligned		29	5	26	887	39	430	26	1,011	84	460
Bases	Total bases		149,750,837	136,807,476	149,750,837	140,695,967	149,750,837	91,083,792	136,807,476	140,695,967	136,807,476	91,083,792
	Aligned	bp	141,467,912	129,748,697	135,095,707	139,499,930	102,512,435	89,901,367	123,572,251	137,934,367	100,065,708	89,599,127
		% for total bases	94.47%	94.84%	90.21%	99.15%	68.46%	98.70%	90.33%	98.04%	73.14%	98.37%
	Unaligned	bp	8,282,925	7,058,779	14,655,130	1,196,037	47,238,402	1,182,425	13,235,225	2,761,600	36,741,768	1,484,665
		% for total bases	5.53%	5.16%	9.79%	0.85%	31.54%	1.30%	9.67%	1.96%	26.86%	1.63%
Syntenic difference	Breakpoints		57,007	53,835	109,568	45,089	116,992	45979	138,441	85,178	118,725	51,951
	Relocations		982	622	219	82	4	48	668	238	68	79
	Translocations		1,980	1,101	19,502	766	28,807	1129	17,489	1,908	28,316	1,629
	Translocations		34	64	445	551	2	39	395	487	4	17
SNP*			29,960		9,655		30,347		24,699		32,729	
INDEL			14,608		17,081		21,267		2,622		14,112	

*Supported by 20 bp over sequence identifer around the detected position

Supplementary Table 5. Predicted gene number and BUSCO completeness of *R. irregularis* DAOM-181602 genomes

	Gene mode (Longest isoform)		Isoform model		Complete BUSCOs (S+D)		Complete and single-copy BUSCOs (S)		Complete and duplicated BUSCOs (D)		Fragmented BUSCOs		Missing BUSCOs	
	#	Average length (AA)	#	Average length (AA)	#	%	#	%	#	%	#	%	#	%
RIR17	41,572	377	43,675	378	273	94.1	243	83.8	30	10.3	11	3.8	6	2.1
RIR17 Non-provisional	27,859	448	29,849	446	273	94.1	243	83.8	30	10.3	11	3.8	6	2.1
JGI_v2.0	26,183	326	-	-	282	97.2	274	94.5	8	2.8	2	0.7	6	2.1
Lin14	27,301	347	29,911	351	256	88.3	186	64.1	70	24.1	7	2.4	27	9.3
JGI_v1.0	30,282	270	-	-	257	88.6	251	86.6	6	2.1	29	10.0	4	1.4

Supplementary Table 6. Number of genes on repetitive regions

	Total gene number	Genes on repetitive reagion	Genes on transposon					Other repetitive region	Genes on non-repetitive region
			LINE	LTR	DNA	unknown	Total transposon		
RIR17	41,572	32,665	3,185	1,735	8,065	15,287	24,185	8,480	8,907
RIR17 Non-provisional gene	27,859	22,455	1,462	1,290	4,354	10,361	15,099	7,356	5,404
JGI_v2.0	26,183	20,106	252	334	1,878	10,043	11,386	8,720	6,077

Supplementary Table 7. Analyzed protein sequences by Orthofinder analaysis

IDs in Figure 1	Species name	Source
Drosophila	*Drosophila melanogaster*	OrthoDB9:Metazoa*
Mus	*Mus musculus* 10090	OrthoDB9:Metazoa*
Mortierella	*Mortierella elongata* AG-77	INSDC:PRJNA196039
R. irregularis	*Rhizophagus irregularis*	RIR17
Phycomyces	*Phycomyces blakesleeanus*	INSDC: PRJNA61391
Rhizopus	*Rhizopus delemar* RA 99-880	OrthoDB9:Fungi*
Aspergillus	*Aspergillus nidulans* FGSC_A4	ASpGD (s10-m04-r06)
Saccharomyces	*Saccharomyces cerevisiae* S288C	Saccharomyces Genome Database
Agaricus	*Agaricus bisporus* var. bisporus	OrthoDB9:Fungi*
Batrachochytrium	*Batrachochytrium dendrobatidis* JAM81	OrthoDB9:Fungi*
Encephalitozoon	*Encephalitozoon cuniculi*	OrthoDB9:Fungi*

*http://www.orthodb.org/?page=filelist

Supplementary Table 8. Blast search of genes involved in the degradation of plant cell wall

Query; Corresponding gene in *Aspergillus niger*, Database; all of the constructed gene models from RIR17 (for blastp search), or RIR17 genomic sequences (for tblastn search)

Activity category in CAzy	CAZy ID	Corresponding gene in *Aspergillus niger* CBS 513.88 (Genbank ID)	Blastp (e-value < 1E-4)	tBlastn (e-value < 1E-4)
		CAK48272	No-hit	No-hit
	PL4	CAK46452	No-hit	No-hit
		CAK48551	No-hit	No-hit
		CAK48529	No-hit	No-hit
		CAK48388	No-hit	No-hit
		CAK47350	No-hit	No-hit
		CAK40523	No-hit	No-hit
Polysaccharide lyases	PL1	CAK37997	No-hit	No-hit
		AAF04492	No-hit	No-hit
	GH7	AAF04491	No-hit	No-hit
		CAK41068	No-hit	No-hit
Cellobiohytdorlases	GH6	CAK39856	No-hit	No-hit
		CAK97324	No-hit	No-hit
		CAK97151	No-hit	No-hit
		CAK46515	No-hit	No-hit
		CAK45495	No-hit	No-hit
		CAK42466	No-hit	No-hit
Lytic polysaccharide mono-		CAK41095	No-hit	No-hit
oxygenases	AA9	CAK38942	No-hit	No-hit
		CAK97324	No-hit	No-hit
		CAK46515	No-hit	No-hit
		CAK44069	No-hit	No-hit
		CAK43558	No-hit	No-hit
		CAK42930	No-hit	No-hit
		CAK42466	No-hit	No-hit
Proteins with cellulose-binding		CAK41068	No-hit	No-hit
motif 1	CBM1	AAF04492	No-hit	No-hit

Supplementary Table 9. Mapped read depth of coverage to RIR17 genes.

	isoform ID	Mean read depth of covrage*	(rDNA) / (single-copy BUSCOs)
BUSCOs	g15690.t1	159.3	-
	g16450.t1	160.3	-
	g24696.t1	157.4	-
	g13527.t1	149.0	-
	g1455.t1	162.1	-
	g11464.t1	153.4	-
	g13372.t1	152.5	-
	g31539.t1	155.3	-
	g6363.t1	146.1	-
	g11459.t1	144.2	-
	Average of ten BUSCOs	154.0	-
rDNAs	rDNA_Consensus_IUPAC	1733.3	11.3

*Calculated by the mapping of DNA short reads (Rir_DNA_PE180) to selected ten BUSCO genes and consensus rDNA sequences from ten rDNA paralogs.

Supplementary Table 10. rDNAs in the previous genome assemblies based on RNAmmer analysis

Genome ID	Assmbly ID	rDNA type	Start	End	Strand	Score	Coresponding gene on RIR17*
JGI_v2.0	scaffold_373	28s_rRNA	3428	8116	-	3249.9	
JGI_v2.0	scaffold_373	18s_rRNA	8228	10038	-	1495.1	C311-1
JGI_v2.0	scaffold_200	18s_rRNA	86443	88253	+	1494.6	
JGI_v2.0	scaffold_200	28s_rRNA	88365	93099	+	3280.4	C312-1
JGI_v2.0	scaffold_17	28s_rRNA	187376	191935	-	3283.5	
JGI_v2.0	scaffold_17	18s_rRNA	192047	193857	-	1494.6	C4-1
JGI_v2.0	scaffold_658	18s_rRNA	20153	21963	+	1494.8	C39-2(Partial)
JGI_v2.0	scaffold_349	18s_rRNA	122313	123445	+	164.4	C39-1(Partial)
JGI_v2.0	scaffold_159	28s_rRNA	45455	46835	+	33.9	C356-1(Partial)
JGI_v1.0	scaffold_3668	28s_rRNA	9	1305	-	252.7	Not analysed
JGI_v1.0	scaffold_3199	18s_rRNA	6	1498	+	956.7	Not analysed
Lin_14	JEMT01001954.1	28s_rRNA	4257	7785	-	3214.9	Not analysed
Lin_14	JEMT01015073.1	18s_rRNA	10	1068	-	134.7	Not analysed
Lin_14	JEMT01012775.1	18s_rRNA	1537	2892	+	845.2	Not analysed

*from mummer alignment

Supplementary Table 11. Mean intragenomic differences among ten rDNA paralogs

Region	Aligned positions	# difference		# indel		Percent identity (%)	
		Average	SD	Average	SD	Average	SD
18SrDNA	1811	1.58	1.09	0.47	0.50	99.91	0.06
ITS1	110	7.13	7.85	2.76	2.54	93.45	7.20
5.8SrDNA	158	5.29	5.14	3.00	3.14	96.65	3.25
ITS2	220	20.51	14.76	8.80	7.72	90.28	6.84
28SrDNA	3548	73.47	48.49	12.29	12.68	97.93	1.37
Whole 48SrDNA cluster	5847	106.13	69.60	27.31	22.84	98.18	1.19

SD = standard deviation

Supplementary Table 12. Detected numbers of tRNA by tRNAscan-SE

	tRNAs decoding Standard 20 AA	Selenocysteine tRNAs (TCA)	Possible suppressor tRNAs (CTA, TTA)	tRNAs with undetermined/ unknown isotypes	Predicted pseudogenes	tRNAs with introns
RIR17	110	0	1	17	12	37
Rhizopus oryzae	237	0	0	0	2	12
Phycomyces blakesleeanus	308	0	1	0	0	12
Mortierella elongata	253	0	0	2	3	12
Abisporus varbisporus	157	0	0	0	3	91
Saccharomyces cerevisiae	286	1	0	2	6	59
