# Supplementary material for: Evidence of non-tandemly repeated rDNAs and their intragenomic heterogeneity in Rhizophagus irregularis
Source: Commun Biol. 2018 Jul 10;1:87. doi: 10.1038/s42003-018-0094-7 (PMC6123716; doi:10.1038/s42003-018-0094-7)
Supplement: Supplementary file 2 — Description of Additional Supplementary Files [file 42003_2018_94_MOESM2_ESM.docx]

**Description of Additional Supplementary Files**

File Name: Supplementary Data 1

Description: Masked repetitive region of RIR17 and related organisms

File Name: Supplementary Data 2

Description: **Gene model confidence based on RNA-seq expression support, homology evidence or protein motif evidence**

"odb9" means the number of orthologous genes on odb9 (The hierarchical catalog of orthologs v9 in orthoDB) based on blast search to odb9 database. "pfam" means the number of determined domains by Pfam search, "RNAseq" means the number of RNAseq library showed >1 RPKM value (33 libraries were analyzed; DRX021574-DRX021597 from DRP002784, and DRX053582-DRX053589 from DRP003319)

File Name: Supplementary Data 3

Description: Gene classification by orthofinder

File Name: Supplementary Data 4

Description: Rapidity expanded/contracted families by CAFE analysis

File Name: Supplementary Data 5

Description: Gene number joined for each orthologous group in orthoDB9

File Name: Supplementary Data 6

Description: Number of RIR17 genes having protein-motif

File Name: Supplementary Data 7

Description: Percentages of the gene on transposable element (raw data for Figure 1d,e)

File Name: Supplementary Data 8

Description: Blast search of "the missing ascomycete core genes orthologs" by Tang et al. 2016

Query; 39 orthologs from Saccharomyces Genome Database (S288C reference), Database; all of the gene models from RIR17 for blastp, and RIR17 genomic sequence for tblastn search

File Name: Supplementary Data 9

Description: Position and size of ribosomal DNAs in RIR17 assemblies

File Name: Supplementary Data 10

Description: Different positions among rDNA paralogs in RIR17
